# Immunocytochemical analysis of P2X2 in rat circumvallate taste buds

**DOI:** 10.1186/1471-2202-13-51

**Published:** 2012-05-23

**Authors:** Ruibiao Yang, Alana Montoya, Amanda Bond, Jenna Walton, John C Kinnamon

**Affiliations:** 1 Department of Biological Sciences, University of Denver, Denver, CO, 80208, USA; 2 Rocky Mountain Taste and Smell Center, Aurora, CO, 80045, USA

## Abstract

**Background:**

Our laboratory has shown that classical synapses and synaptic proteins are associated with Type III cells. Yet it is generally accepted that Type II cells transduce bitter, sweet and umami stimuli. No classical synapses, however, have been found associated with Type II cells. Recent studies indicate that the ionotropic purinergic receptors P2X2/P2X3 are present in rodent taste buds. Taste nerve processes express the ionotropic purinergic receptors (P2X2/P2X3). P2X2/P2X3^Dbl−/−^ mice are not responsive to sweet, umami and bitter stimuli, and it has been proposed that ATP acts as a neurotransmitter in taste buds. The goal of the present study is to learn more about the nature of purinergic contacts in rat circumvallate taste buds by examining immunoreactivity to antisera directed against the purinergic receptor P2X2.

**Results:**

P2X2-like immunoreactivity is present in intragemmal nerve processes in rat circumvallate taste buds. Intense immunoreactivity can also be seen in the subgemmal nerve plexuses located below the basal lamina. The P2X2 immunoreactive nerve processes also display syntaxin-1-LIR. The immunoreactive nerves are in close contact with the IP_3_R3-LIR Type II cells and syntaxin-1-LIR and/or 5-HT-LIR Type III cells. Taste cell synapses are observed only from Type III taste cells onto P2X2-LIR nerve processes. Unusually large, “atypical” mitochondria in the Type II taste cells are found only at close appositions with P2X2-LIR nerve processes. P2X2 immunogold particles are concentrated at the membranes of nerve processes at close appositions with taste cells.

**Conclusions:**

Based on our immunofluorescence and immunoelectron microscopical studies we believe that both perigemmal and most all intragemmal nerve processes display P2X2-LIR. Moreover, colloidal gold immunoelectron microscopy indicates that P2X2-LIR in nerve processes is concentrated at sites of close apposition with Type II cells. This supports the hypothesis that ATP may be a key neurotransmitter in taste transduction and that Type II cells release ATP, activating P2X2 receptors in nerve processes.

## Background

The rodent taste bud is a collection of approximately 50–150 spindle-shaped cells, termed taste cells. A typical taste bud extends from the basal lamina to the taste pore, where apical microvilli extend into the oral cavity and interact with sapid molecules [1,2]. Murine taste buds contain three primary differentiated cell types: Type I (“dark” cells), Type II (“light”, or “receptor” cells) and Type III (“presynaptic”) cells (for review see [3]). Type I cells are the most abundant cell type and are characterized by the presence of prominent invaginations in the electron-dense nuclei and membrane-bound granules in the apical cytoplasm. Type I cells possess several long, slender microvilli that extend into the taste pore. Type I cells have sheet-like membrane extensions that envelop other cells in a glia-like manner and have been reported to express blood group antigen H [4,5]. Type I cells may be responsible for the “pore substance” released into the taste pore [6]. The ecto-ATPase, NTPDase2, is present on the plasma membranes of Type I cells [7], which may function in the degradation of ATP [8]. Vandenbeuch et al. [9] have reported the presence of Amiloride-sensitive Na^+^ channels in Type I cells. Type II (receptor) cells have ovoid to circular nuclear profiles, electron-lucent cytoplasm and prominent smooth endoplasmic reticulum in the supranuclear cytoplasm. Type II cells possess short, brush-like microvilli of the same length and express the immunocytochemical markers IP_3_R3 [10] and PLCβ2 [11]. The G-protein, α-gustducin, is also present in a subset of Type II cells [12,13]. There are two non-overlapping subsets of Type II cells. One subset expresses the G protein, a-gustducin, while the other subset expresses protein gene product 9.5 (PGP 9.5). Type II cells express combinations of taste receptor proteins for detecting sweet (T1R2/T1R3), bitter (T2R) and umami (T1R1/T1R3). Type II cells that signal sweet taste express Gα14 [14]. Although Type II cells detect bitter, sweet, and umami, they do not form classical synapses onto nerve fibers [11,13,15-18]. Type III (“presynaptic”) cells are intermediate in morphology between Type I and Type II cells. Type III cell cytoplasm is electron-lucent like a Type II cell but the nuclei are slender with prominent invaginations like Type I cells. A Type III cell possesses a single, large, blunt, apical microvillus. Type III cells also possess numerous, small clear vesicles and a variable amount of large dense-cored vesicles associated with classical synapses onto nerve processes. Murray [19] proposed that the Type III cell was the gustatory receptor cell. The Type III cell has been termed the *output* or *presynaptic cell* in taste buds [20,21]. Rodent Type III cells display immunoreactivity to neural cell adhesion molecule (NCAM), serotonin (5-HT) and the synaptic proteins SNAP-25, synaptobrevin-2, syntaxin-1, and synaptophysin [18,22-27]. Non-overlapping subsets of Type III cells display immunoreactivity to serotonin or protein gene product 9.5 [28]. Recent studies indicate that Type III cells release 5-HT upon gustatory stimulation [29-31]. The candidate sour taste receptor, PKD2L1, is present in taste buds [32] and is expressed by Type III taste cells [33].

Our Laboratory has shown that classical synapses and the synaptic SNARE proteins SNAP-25, synaptobrevin-2 and syntaxin-1 are associated with Type III cells [18,26,27]. No classical synapses have been found associated with Type II cells. Nevertheless, it is generally accepted that Type II cells transduce bitter, sweet and umami [10,11,13,34-36]. A recent major finding in taste signal transduction provides evidence that ATP serves as a neurotransmitter released from taste cells to nerve processes in taste buds [16]. Extracellular ATP signaling acts through two families of receptors: P2X ionotropic ligand-gated ion channel receptors (P2X1-P2X7) and P2Y metabotropic ligand-gated ion channel receptors (P2Y1-P2Y8) [37-40].

Evidence supports a major role for P2X2 and P2X3 subunits in mediating the transduction of ATP [38]. Taste nerves express the ionotropic ATP-gated P2X2 and P2X3 receptors in rodent taste buds [41-43]. ATP was first shown to be a neurotransmitter in rodent taste buds when Finger et al. [16] showed that ATP was released in response to gustatory stimuli. In addition, they showed that responses to bitter, sweet and umami stimuli were eliminated in P2X2/P2X3^Dbl−/−^ mice. P2X2/P2X3^Dbl−/−^ mice have also been shown not to taste NaCl or the artificial sweetener SC45647 [44]. Residual chemosensing abilities to detect bitter and sweet stimuli are believed to be mediated by postingestive detection [45]. Kataoka et al. [43] observed expression of P2X3 receptors in intragemmal nerve processes of the rat. Ishida et al. [42] found that P2X2- and P2X3-LIR fibers in fungiform papillae originate from the chorda tympani nerve. P2X2 and P2X3 receptors are also present in rat trigeminal ganglion neurons [46]. Recently, Hayato et al. [47] reported that P2X2 and P2X7 receptors are expressed on Type II and Type III taste bud cells of the mouse.

Studies describing the non-vesicular release of ATP within taste buds were published by Huang et al. [48] and Romanov et al. [49]. Romanov et al. [49] proposed that a non-vesicular release of ATP occurs through connexin-43 hemichannels, while Huang et al. [48] proposed that ATP is released through pannexin-1 hemichannels. Huang et al. [29] have also proposed autocrine and paracrine roles for ATP and serotonin in mouse taste buds. Release of ATP from Type II cells onto Type III cells is thought to elicit the release of serotonin by Type III cells. This serotonin release then provides negative feedback onto the Type II cells, resulting in decreased release of ATP.

In the present study we have used immunofluorescence and immunoelectron microscopy to demonstrate that: 1) Most all intragemmal nerve processes display P2X2-like immunoreactivity (−LIR), 2) Type III cells form conventional synaptic contacts onto P2X2-LIR nerve processes, 3) P2X2 receptors are distributed in a punctate manner adjacent to contacts with Type II cells, 4) Large, atypical mitochondria with tubular cristae are found specifically in Type II cells at sites of close apposition with nerve processes.

## Results

**Figure 1:**
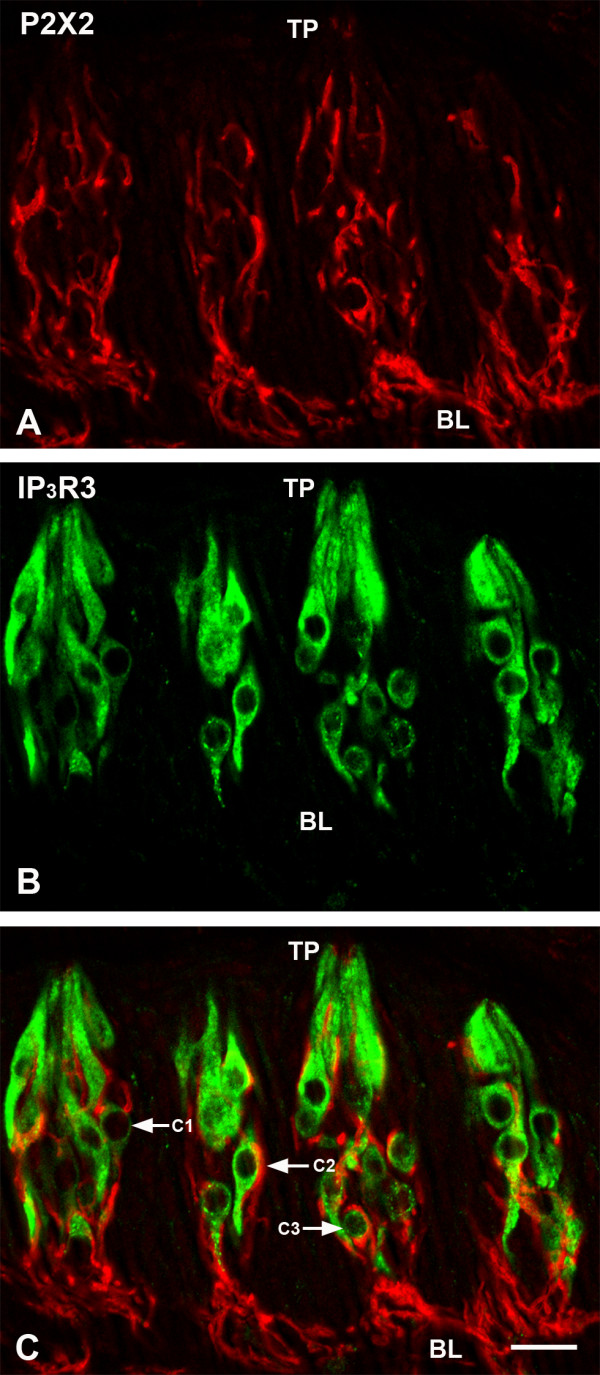
**Double-label confocal images showing P2X2- with IP**_**3**_**R3-LIR in rat circumvallate taste buds. A:** P2X2-LIR is present in nerve processes. Intense immunoreactivity can be seen in the nerve plexus near the basal lamina (BL). **B:** Type II taste cells displaying IP_3_R3-LIR. **C.** Double-label image showing P2X2-LIR nerve processes in close apposition with IP_3_R3-LIR taste cells (C1 and C2). C3 indicates an IP_3_R3-LIR cell that is enveloped by a P2X2-LIR nerve process. TP: taste pore, Scale bar = 20 μm.

P2X2-LIR is present in intragemmal nerve processes in rat circumvallate taste buds (Figure 1A). The P2X2 immunoreactive nerve processes ramify throughout the entire taste bud, extending apically from the basal lamina almost to the taste pore. Intense immunoreactivity can also be seen in the subgemmal nerve plexuses located below the basal lamina. However, P2X2-LIR was not observed in the non-gustatory lingual epithelium surrounding taste buds.

### P2X2 and IP_3_R3

IP_3_R3 immunoreactivity is commonly used as a marker for Type II cells. IP_3_R3-LIR cells have large, circular nuclei, a distinguishing feature of Type II cells (Figure 1B). The immunoreactivity is cytoplasmic only; nuclei display no staining. Cytoplasmic immunoreactivity extends evenly throughout the cytoplasm from the basal lamina to the taste pore. P2X2-LIR nerve processes are often in close apposition with IP_3_R3-LIR cells. Some of these contacts are in the form of discrete terminal puncta, as shown in Figure 1C (cell #1, C1). This particular cell is contacted just apical to the nuclear region by two small nerve processes that end as swollen terminals. Cell #2 (C2) has a large surface in contact with a nerve process. At the other extreme, some IP_3_R3-LIR cells are virtually enveloped by P2X2-LIR nerve processes (cell #3, C3).

### P2X2 and Syntaxin-1

**Figure 2:**
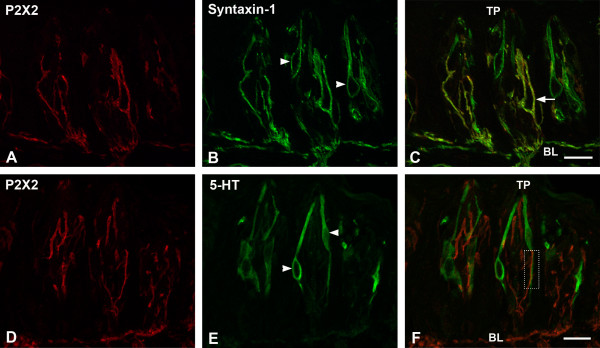
**Double-label confocal images showing P2X2- with syntaxin-1- or 5-HT-LIR in rat circumvallate taste buds. A:** P2X2-LIR nerve processes extend from basal lamina to taste pore. **B:** A subset of taste cells (arrowheads) and nerve processes in taste buds displaying syntaxin-1-LIR. **C:** P2X2-LIR nerve processes also express syntaxin-1 (arrow). **D:** P2X2-LIR. **E:** 5-HT is present in a subset of taste cells (arrowheads). No colocalization occurs between P2X2- and 5-HT-LIR in the taste buds, however, P2X2 immunoreactive nerve processes are in close apposition with 5-HT-LIR cells (e.g., rectangular box). TP: taste pore; BL: basal lamina. Scale bars = 20 μm.

Syntaxin-1, the t-SNARE protein, has been demonstrated to be a cell marker for Type III cells as well as nerve processes [27]. Double labeling of P2X2 (Figure 2A) with syntaxin-1 (Figure 2B) shows syntaxin-1-LIR is present in the Type III taste cells and both subgemmal and intragemmal nerve processes. All syntaxin-1-LIR nerve processes also display P2X2-LIR and vice versa. We did not find any syntaxin-1-LIR taste cells expressing P2X2-LIR (Figure 2C), but the cells immunoreactive for syntaxin-1 are in close contact with P2X2-LIR nerve processes, suggesting that P2X2-LIR nerve processes are in close contact with Type III cells.

### P2X2 and Serotonin (5-HT)

Experiments using P2X2 (Figure 2D) and 5-HT (Figure 2E), a known cell marker to be present in a subset of Type III cells [28], were performed to determine if P2X2-LIR nerve processes contact this subset of Type III cells. 5-HT-LIR (Figure 2E) is present in a small subset of taste cells [28]. Serotonin (5-HT)-LIR cells are narrow and fusiform shaped, typical of Type III cells. Immunoreactivity in the cytoplasm extends from the basal lamina to the taste pore, and is present only in taste cells. 5-HT–LIR cells resemble syntaxin-1-LIR cells in both shape and structure, and have been shown to colocalize with a subset of syntaxin-1-LIR (Type III) cells. No colocalization occurs between 5-HT-LIR and P2X2-LIR (Figure 2F), and no 5-HT immunoreactivity is present in the nerve processes. All nerve processes that are in close apposition with Type III cells display P2X2-LIR (Figures 2D and 2F).

### P2X2-LIR nerve processes

**Figure 3:**
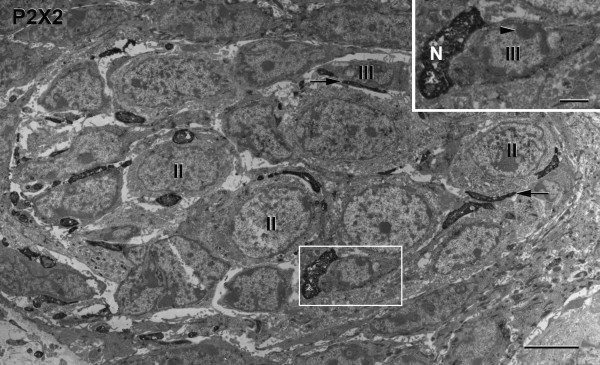
**A DAB immunoelectron micrograph demonstrates that P2X2-LIR is present in nerve processes in a transverse section of a taste bud.** The P2X2 immunoreactive nerve processes (arrows) are in close apposition with Type II (II) and Type III (III) taste cells. **Inset:** A P2X2-LIR nerve process (N) closely contacts a Type III taste cell (III); its nucleus contains characteristic electron-dense patches of heterochromatin (arrowhead). Scale bar = 2 μm; Scale bar = 0.5 μm in **Inset**.

Immunoelectron microscopy using diaminobenzidine (DAB) demonstrates that P2X2 is present in nerve processes (Figure 3). All of the intragemmal nerve processes that we observed in rat circumvallate taste buds display P2X2-LIR. P2X2-LIR nerve processes are in close apposition with both Type II and Type III taste cells. Type II taste cells have numerous mitochondria found adjacent to swollen smooth endoplasmic reticulum. These cells form close contacts with P2X2-LIR nerve processes. The taste cell shown in the inset for Figure 3 contains electron-dense patches of heterochromatin in the nucleus, which is a distinguishing feature of Type III cells. Regular mitochondria are often found in abundance within the P2X2-LIR nerve processes.

### Synapses

**Figure 4:**
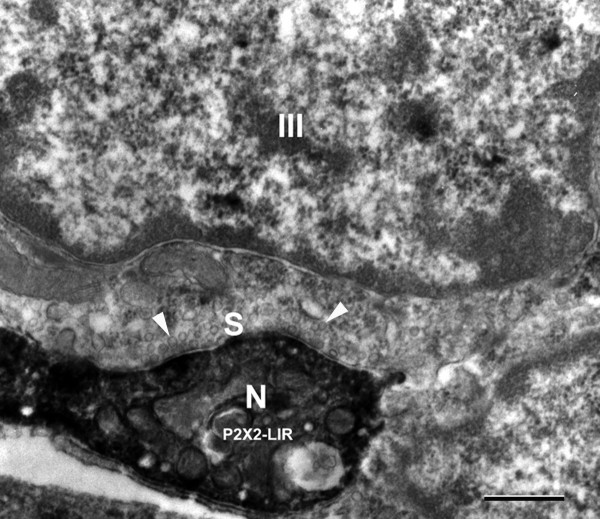
**A DAB immunoelectron image showing a Type III cell (III) with a synaptic contact (S) onto a P2X2 immunoreactive nerve process (N).** Numerous small clear synaptic vesicles (arrowheads) are present in presynaptic zone. Scale bar = 0.5 μm.

Figure 4 shows a conventional synapse from a Type III cell onto an electron-dense P2X2-LIR nerve process. The synapse consists of parallel, apposed membranes with a cleft between the Type III cell and the P2X2-LIR nerve process. Numerous small, clear synaptic vesicles are present at the presynaptic zone. Some of these vesicles are docked at the presynaptic membrane, but most are in the presynaptic cytoplasm. The postsynaptic P2X2-LIR nerve process contains numerous, small mitochondria. Such synapses were only observed from Type III taste cells onto P2X2-LIR nerve processes. No synapses were observed from Type II cells onto the P2X2-LIR nerve processes.

### Atypical Mitochondria in Type II cells

**Figure 5:**
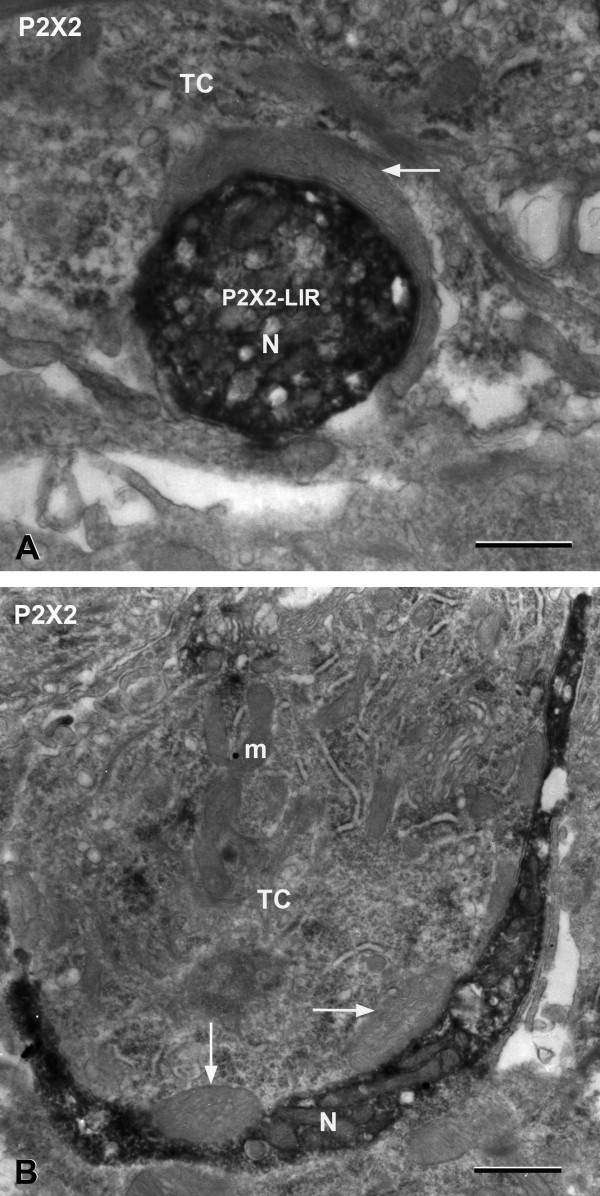
**DAB immunoelectron micrographs showing Type II cells (TC) with “atypical” mitochondria located only at close appositions with P2X2 immunoreactive nerve processes. A:** A P2X2-LIR nerve process (N) is partially enveloped by a large “atypical” mitochondrion (arrow). **B:** A Type II cell containing two large “atypical” mitochondria (arrows) that are in contact with a P2X2-LIR nerve process (N). m: regular mitochondrion. Scale bars = 0.5 μm.

No conventional synapses were observed from Type II taste cells onto nerve processes. We did observe, however, that Type II cells often contained large, “atypical” mitochondria that were located only at close appositions between the membranes of Type II cells and P2X2-LIR nerve processes (Figure 5). The cristae of the atypical mitochondria exhibit a “twisted-energized” or “swollen twisted-energized configuration” [50-53], resembling electron-dense sacs or tubules within the mitochondrion (Figures 5A and B). This contrasts with the typical mitochondria distributed throughout the cytoplasm of Type II taste cells, which are elongate, slender structures rarely exceeding 0.3 μm in width and possessing lamellar or baffle cristae (e.g., “m” in Figure 5B). The “atypical” mitochondria that we have observed have diameters two to three times larger than conventional mitochondria. The atypical mitochondria are separated from the cytoplasmic leaflet of the taste cell membrane by a gap of approximately 20 nm (Figure 5A).

### Immunogold microscopy

**Figure 6:**
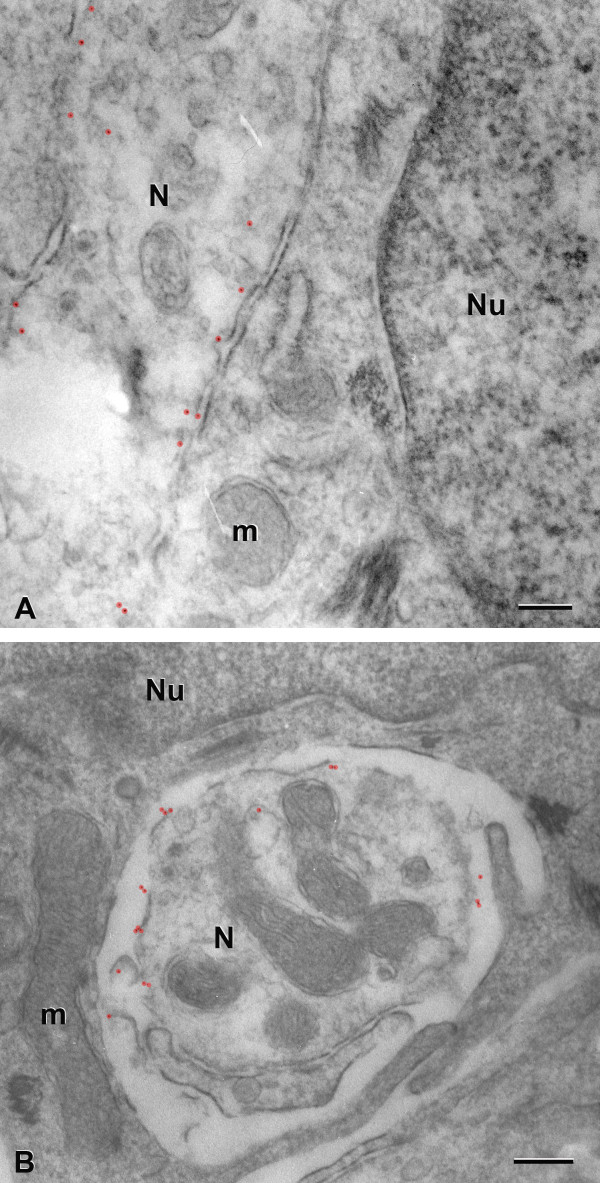
**Immunogold micrographs showing that 6 nm gold particles are found only at the junctions between Type II cells and nerve processes.** The colloidal gold particles are marked by transparent red dots. **A and B:** Gold particles are concentrated near the membrane of nerve processes (N) in close apposition with Type II cells (Nu). m: mitochondria. Scale bars = 0.2 μm.

Our results indicate that P2X2 receptors are concentrated at the junctions between Type II cells and nerve processes in a punctate manner (Figure 6). Since 6 nm immunogold particles were used in this research, we took pictures with a magnification of 30,000. The immunogold particles are very small, hence we masked each gold particle with a transparent red dot using Photoshop. The signal is very specific, being located near the membrane of nerve processes in close apposition with Type II cells (Figures 6A and B). Virtually no background immunoreactivity for P2X2 was observed. No immunogold particles were found at the synapses between Type III cells and nerve processes.

## Discussion

P2X2/3 receptors in intragemmal nerve processes were first reported by Bo et al. in 1999 [41]. Finger et al. [16] found that P2X2/3 double knockout mice are not responsive to sweet, umami and bitter stimuli, and proposed that ATP acts as a neurotransmitter in taste buds. In a previous study [54], we used conventional electron microscopy to compare taste bud ultrastructure in P2X2/3 double knockout mice with taste buds in wild-type mice. There were no differences at the ultrastructural level between P2X2/3 double knockout mice and the wild-type mice taste buds. Taste cells and nerve processes in the P2X2/3 double knockout mouse taste buds show normal ultrastructural features. Based on our present fluorescence and immunoelectron microscopical studies we believe that both perigemmal and most, if not all intragemmal nerve processes display P2X2-LIR. Numerous immunoreactive nerve processes can be seen coursing throughout the lamina propria as well as several immunoreactive nerve processes that appear to be projecting from the subgemmal plexuses through the basal lamina into the taste buds. All P2X2-LIR nerve processes display syntaxin-1-LIR. Our immunoelectron microscopical results show that the P2X2-LIR nerve processes contain numerous mitochondria. These nerve processes are in close apposition with Type II or Type III cells, and some immunoreactive nerve processes even envelop much of the taste cells. Using diaminobenzidine (DAB) immunoelectron microscopy, P2X2-LIR appears to be distributed homogeneously throughout the nerve processes. Using colloidal gold immunoelectron microscopy, however, the gold particles were localized on the nerve process membranes at close appositions with the taste cell membranes.

Type II cells are in intimate contact with P2X2-LIR intragemmal nerve processes, providing a pathway for functional connectivity between Type II cells and P2X2-LIR nerve processes. This is best shown in Figure 1C, which shows an IP_3_R3-LIR Type II cell (C1) with contacts onto two expanded terminals of a P2X2-LIR nerve process. The results were similar when we colocalized P2X2-LIR with the Type III cell markers, 5-HT [24] and syntaxin-1 [27]. Although the Type III cells with 5-HT and/or syntaxin-1-LIR did not display P2X2-LIR, these Type III cells are in intimate contact with P2X2 immunoreactive nerve processes. In the present study, we did not find taste cells in rat circumvallate taste buds displaying P2X2-LIR. However, Hayato et al., [47] indicated that a subset of Type III taste cells in mouse fungiform taste buds express P2X2-LIR. Based on our data from confocal microscopy, DAB immunoelectron and immunogold microscopy, P2X2 is only present in nerve processes in rat circumvallate taste buds. We did not find any taste cells displaying P2X2-LIR. Although some P2X2-LIR in Figure 1A appears to be in a taste cell, the colocalization results in Figure 1C demonstrate that the P2X2-LIR was present in a nerve process that enveloped an IP_3_R3-LIR taste cell (C3, Figure 1C). Another possibility is that these differences in results may arise from differences between rat and mouse taste buds. We have previously observed that some antibodies show different immunoreactivity patterns in rat and mouse taste buds, or even different immunoreactivity patterns between different papillae of the same species [55].

In our previous studies [18,26,27], taste cell synapses were found to be associated only with Type III cells. In the present study we found that all of the postsynaptic nerve processes that we observed displayed P2X2-LIR. It is significant that Type III cells form synaptic contacts containing small, clear vesicles and large, dense-cored vesicles onto P2X2-LIR nerve processes*.* In addition, our results indicates that Type III cells express pannexin-1-LIR [56]. Thus, Type III cells may release transmitters from small, clear vesicles and/or large, dense-cored vesicles onto the P2X2-LIR nerve fibers, and Type III cells might also release ATP onto nerve processes and/or other taste cells.

Most mitochondria in taste cells are long, slender structures rarely exceeding 0.3 μm in diameter [52]. The atypical mitochondria that we have observed have diameters two to three times larger than conventional mitochondria. Typically the cristae of the atypical mitochondria exhibit a “twisted-energized” or “swollen twisted-energized configuration” [50,51,53], resembling electron-dense sacs or tubules within the mitochondrion. The outer membranes of these atypical mitochondria are closely apposed to the cytoplasmic leaflet of the Type II taste cell’s membrane (Figure 5).

What are the roles of the atypical mitochondria in taste cell function? Based on their location at the sites of close apposition between Type II cells and nerve processes, we speculate that the atypical mitochondria may be the source of ATP that is released through the pannexin/connexin hemichannels onto nerve processes. Approximately 94% of the ATP synthase in a mitochondrion is located in the cristal membrane [57]. Increases in the ratio of cristal membrane to inner membrane is associated with increases in oxidative phosphorylation and the production of ATP [58]. Because of their large size, atypical mitochondria at taste cell-nerve fiber contacts may contain significant amounts of ATP. Thus the atypical mitochondria we have observed may be ideal sources of ATP for use as a neurotransmitter at these contacts.

Another possibility is that mitochondria may modulate the open/closed state of pannexin/connexin hemichannels by a regulated uptake and release of Ca^2+^, which is known to modulate the coupling coefficient of gap junctions. Support for this idea comes from studies which have shown that mitochondria can take up and release Ca^2+^ in response to GPCR activation via the IP3 pathway [59,60]. The IP3 pathway is present in Type II taste cells [11,15] and is required for bitter, sweet, and umami transduction [61]. In addition, the opening of pannexin hemichannels has been reported to be controlled by Ca^2+^[48]. Thus, whether the atypical mitochondria are the source of ATP, regulate ATP release through Ca^2+^, or both, the close apposition of the unusually large mitochondria with pannexin hemichannels and nerve fibers containing P2X2 receptors provides a potential pathway for the control of ATP release.

## Conclusions

P2X2-like immunoreactivity is present in intragemmal nerve processes in rat circumvallate taste buds. The immunoreactive nerves are in close contact with the IP_3_R3-LIR Type II cells, syntaxin-1-LIR and/or 5-HT-LIR Type III cells. Taste cell synapses are observed only from Type III taste cells onto P2X2-LIR nerve processes. Large, “atypical” mitochondria in the Type II taste cells are found only at close appositions with P2X2-LIR nerve processes. P2X2 immunogold particles are concentrated at the membranes of nerve processes at close appositions with taste cells. Based on our immunofluorescence and immunoelectron microscopical studies, we suggest that ATP may be a key neurotransmitter in taste transduction, and Type II cells may release ATP to activate the P2X2 receptors in nerve processes.

## Methods

Adult Sprague–Dawley male rats (250–350 g) were used for these studies. Animals were cared for and housed in facilities approved by the Institutional Animal Care and Use Committee of the University of Denver. For the colocalization of P2X2 with serotonin, animals were injected with 5-hydroxytryptophan (5-HTP, 80 mg/kg, i.p.) one hour before they were perfused. All rats were anesthetized with a mixture of sodium ketamine (200 mg/kg) and xylazine (70 mg/kg) (i.p.). Primary and secondary antibodies used for these studies are listed in Tables 1 and 2.

**Table 1:** Primary antibodies

**Antibodies**	**Species**	**Dilution**	**Source**	**Code no.**
P2X2	Rabbit	1:100	Alomone	APR-003
IP_3_R3	Mouse	1:100	Transduction Lab.	610313
Syntaxin-1	Mouse	1:100	Sigma	S0664
Serotonin	Mouse	1:100	Abcam	ab16007

**Table 2:** Secondary antibodies

**Antibodies**	**Dilution**	**Source**	**Code no.**
Cyanine (Cy5) goat anti-rabbit IgG	1:100	Jackson Lab	111-175-144
Fluorescein (FITC) goat anti-mouse IgG	1:50	Jackson Lab.	115-095-100
Biotin-conjugated goat anti-rabbit IgG	1:200	Jackson Lab.	111-065-144
6 nm colloidal gold goat anti-rabbit IgG	1:20	Jackson Lab.	111-195-144

### P2X2

Polyclonal antibody anti-P2X2 receptor was raised from rabbit against the peptide (C)SQQD STSTD PKGLA QL, corresponding to residues 457–472 of rat P2X2, located at intracellular C-terminus. Western blot showed a specific band of expected size at 75 kDa (Alomone Labs, manufacture’s technical information) [16].

### IP_3_R3

The monoclonal antibody, IP_3_R3 is raised against an immunogen corresponding to amino acides 20–230 of human IP_3_R3. Western blot showed a specific band of expected size at 300 kDa (BD Transduction Lab., manufacturer’s technical information) [10].

### Syntaxin-1

Monoclonal anti-syntaxin clone HPC-1 (mouse IgG1 isotype) was raised against a synaptosomal plasma membrane fraction from adult rat hippocampus [62] and recognizes an epitope of HPC-1 antigen in the cytoplasmic surface of plasma membrane. Western blot showed a specific band of expected size at 35 kDa (Sigma, manufacturer’s technical information) [27].

### 5-HT

Serotonin (5-HT) antiserum was generated in a rabbit against serotonin coupled to bovine serum albumin with paraformaldehyde. This antibody was quality control tested using standard immunohistochemical methods (manufacturer’s technical information) [63].

### Immunohistochemistry for confocal microscopy

Fifteen rats were perfused for 10 seconds through the left ventricle with 0.1% sodium nitrite, 0.9% sodium chloride and 100 units sodium heparin in 100 ml 0.1 M phosphate buffer (pH 7.3). This was followed by perfusion fixation in 4% paraformaldehyde in 0.1 M phosphate buffer for 10 minutes. All perfusates were warmed to 42°C before use. After perfusion the excised circumvallate papillae were fixed in fresh fixative for 3 hours at 4°C. The tissues were cryoprotected with 30% sucrose in 0.1 M phosphate buffer overnight at 4°C.

### Single labeling

Cryostat sections from three rats (20 μm thick, Microtome Cryostat HM 505E, MICROM, Laborgeräte, Germany) containing circumvallate taste buds were blocked in 5% normal goat serum and 0.3% Triton X-100 in 0.1 M phosphate buffered saline (PBS) (pH 7.3) for one hour at room temperature, then incubated in primary antibody polyclonal anti-P2X2 receptor in 0.1 M PBS (pH 7.3) overnight at 4°C. After washing, the sections were exposed to affinity-purified secondary antibody Cy5-conjugated to goat anti-rabbit IgG in 0.1 M PBS (pH 7.3) for one hour at room temperature.

### Double labeling

Cryostat sections (20 μm thick) from twelve rat circumvallate taste buds were blocked in 5% normal goat serum, 1% BSA, and 0.3% Triton X-100 in 0.1 M PBS (pH 7.3) for one hour at room temperature. If the primary antibody to IP_3_R3 was used, the sections were processed for antigen retrieval by incubation with 10 mM sodium citrate (pH 9) for 30 minutes at 80°C prior to blocking. The sections were incubated in a combination of two primary antibodies: rabbit polyclonal antibody P2X2 with one of the following monoclonal antibodies, IP_3_R3 (4 rats), syntaxin-1 (4 rats) or serotonin (4 rats) in 0.1 M PBS (pH 7.3) overnight at 4°C. After rinsing in 0.1 M PBS for 30 minutes, the sections were treated in a cocktail of two secondary antibodies consisting of Cyanine (Cy5)-conjugated to goat anti-rabbit IgG and Fluorescein (FITC) conjugated to goat anti-mouse IgG in 0.1 M PBS for one hour at room temperature.

The sections were mounted on Superfrost Plus microscope slides (Fisher Scientific Inc.) and coverslipped using antifade Fluorogel (Electron Microscope Sciences, Hatfield, PA). A Zeiss Axioplan-2 fluorescence microscope (Zeiss, Germany) was used to acquire the images using an Apotome confocal attachment and AxioVision software. Sequential acquisition was used on double-label sections to avoid excitation of inappropriate fluorophores. All confocal images in Figures 1 and 2 are single optical images that were prepared for publication with Adobe Photoshop software (CS, Adobe Systems, Mountain View, CA) adjusting only brightness and contrast.

### Controls

Primary antibodies were excluded from the processing to check for any species related cross-reactivity. Elimination of one of the primary antibodies with application of both secondary antibodies confirmed secondary antibody specificity. No immunoreactivity was observed under these conditions. A preabsorption control with peptide (P2X2_457-472_ peptide, Lot# APR003AG0540, Alomone Labs) was performed to test the specificity of the P2X2 antibody. The final concentration 1 μg peptide per 1 μg antibody was used in this study and no immunoreactivity was found in the preabsorption control.

### Diaminobenzidine (DAB) electron microscopy

Eight rats were perfused as for immunohistochemistry. After perfusion, the excised circumvallate papillae were fixed in fresh fixative for 3 hours at 4°C. Sections (80 μm thick) were sliced with a vibratome (Vibratome Series 1000, Ted Pella Inc., Redding, CA) and then blocked with 2% normal goat serum, 3% BSA and 0.1% fish gelatin in 0.1 M PBS (pH 7.3) for one hour at 4°C. This was followed by incubation with the primary antibody P2X2 in 0.1 M PBS for 24 hours at 4°C. After rinsing in 0.1 M PBS, the sections were incubated in affinity purified biotinylated goat anti-rabbit IgG secondary antibody in PBS for two hours at room temperature. The sections were incubated with avidin-biotin complex (Elite Vectastain, Vector Laboratories Inc., Burlingame, CA) in PBS for one hour at room temperature. After rinsing, sections were treated for 10 minutes in 0.05 M Tris buffer (pH 7.3) containing 0.05% DAB. The label was visualized by floating the sections for 2–4 minutes in a fresh aliquot of the above DAB mixture that had been activated with hydrogen peroxide to a final concentration of 0.002%.

Sections were washed 3X5 minutes in 0.1 M phosphate buffer (pH 7.3) and postfixed in 1% OsO_4_ in 0.1 M phosphate buffer for 15 minutes. After rinsing in 0.05 M sodium maleate buffer (pH 5.2), the sections were stained en bloc in 1% uranyl acetate in 0.025 M sodium maleate buffer (pH 6.0) for overnight at 4°C, followed by dehydration in an alcohol series, processed through propylene oxide, and embedded with Eponate 12 (Ted Pella Inc., Redding, CA). The sections were re-embedded using the technique of Crowley and Kinnamon [64]. Thin sections (90–120 nm) were cut with a diamond knife on a Reichert Ultracut E or Leica Ultracut UCT ultramicrotome. These thin sections were examined with a HITACHI H-7000 transmission electron microscope at 75 kV. Controls consisted of omitting the primary antiserum, secondary antibody, and biotin-avidin complex respectively. No immunoreactivity was observed under these conditions.

### Pre-embedding colloidal gold microscopy

Twelve rats were perfused with 4% paraformaldehyde and 0.05% glutaraldehyde in 0.1 M phosphate buffer. After perfusion, the excised circumvallate papillae were fixed in fresh fixative for 3 hours at 4°C. Sections (80 μm thick) were sliced with a vibratome and blocked for one hour at 4°C. This was followed by incubation with P2X2 primary antiserum in 0.1 M PBS for 24 hours at 4°C. After rinsing in PBS, the sections were incubated in 6 nm colloidal gold-affinipure goat anti-rabbit IgG in PBS for two hours at room temperature. Sections were processed and embedded for electron microscopy. Thin sections (90–120 nm) were cut with a diamond knife on a Reichert Ultracut E or Leica Ultracut UCT ultramicrotome, and examined with a HITACHI H-7000 transmission electron microscope at 75 kV. Controls consisted of omitting the primary antiserum. No immunoreactivity was observed under these conditions.

## Abbreviations

5-HT: 5-hydroxytryptamine (serotonin); IP_3_: Inositol 1,4,5-trisphosphate; IP_3_R3: Inositol 1,4,5-trisphosphate receptor type 3; PLCβ2: Phospholipase C beta 2; GPCR: G protein-coupled receptor.

## Authors’ contributions

RY and JCK conceived the study, designed and executed most of the experiments, and participated in data interpretation and in manuscript preparation. AM, AB, and JW conducted partial experiments and provided technical support. JW conducted the experiments described in Figure 2. All authors have read and approved the final manuscript.
